# Manufacturing and Characterisation of Polymeric Membranes for Water Treatment and Numerical Investigation of Mechanics of Nanocomposite Membranes

**DOI:** 10.3390/polym13101661

**Published:** 2021-05-20

**Authors:** Seren Acarer, İnci Pir, Mertol Tüfekci, Güler Türkoğlu Demirkol, Neşe Tüfekci

**Affiliations:** 1 Faculty of Engineering, Department of Environmental Engineering, İstanbul University-Cerrahpaşa, Avcılar Kampüsü, İstanbul 34320, Turkey; gulertde@iuc.edu.tr (G.T.D.); nese@iuc.edu.tr (N.T.); 2 Faculty of Mechanical Engineering, İstanbul Technical University, İstanbul 34437, Turkey; pirin@itu.edu.tr; 3 Department of Mechanical Engineering, Imperial College London, South Kensington Campus, Exhibition Road, London SW7 2AZ, UK

**Keywords:** finite element, membrane, Mori–Tanaka homogenisation, nanocomposite

## Abstract

In this study, polyethersulfone (PES) and polyvinylidene fluoride (PVDF) microfiltration membranes containing polyvinylpyrrolidone (PVP) with and without support layers of 130 and 150 μm thickness are manufactured using the phase inversion method and then experimentally characterised. For the characterisation of membranes, Fourier transform infrared spectroscopy (FTIR), scanning electron microscopy (SEM), and pore size analysis are performed, the contact angle and water content of membranes are measured and the tensile test is applied to membranes without support layers. Using the results obtained from the tensile tests, the mechanical properties of the halloysite nanotube (HNT) and nano-silicon dioxide (nano SiO_2_) reinforced nanocomposite membranes are approximately determined by the Mori–Tanaka homogenisation method without applying any further mechanical tests. Then, plain polymeric and PES and PVDF based nanocomposite membranes are modelled using the finite element method to determine the effect of the geometry of the membrane on the mechanical behaviour for fifteen different geometries. The modelled membranes compared in terms of three different criteria: equivalent stress (von Mises), displacement, and in-plane principal strain. Based on the data obtained from the characterisation part of the study and the numerical analysis, the membrane with the best performance is determined. The most appropriate shape and material for a membrane for water treatment is specified as a 1% HNT doped PVDF based elliptical membrane.

## 1. Introduction

Polymers such as polyethersulfone (PES), polysulfone (PSf), polyvinylidene fluoride (PVDF), polyacrylonitrile (PAN) and aromatic and aliphatic polyamides (PA) are widely used in the preparation of water treatment membranes due to their superior physical and chemical properties, low cost and they are easily manufactured [1,2,3,4,5]. Alongside the need to increase the selectivity, permeability, chemical and thermal stability of polymeric membranes, it is still necessary to provide adequate mechanical stability to those membranes [6]. The mechanical stiffness and strength of the polymeric membranes are not only important in the structural design of the membrane system, but also in the prediction of the reliability and service life of the membrane. The mechanical stiffness and strength of the membranes are affected by fouling, biological growth, backwashing, chemical treatment, and ageing [7]. In systems with mechanically damaged membranes, the quality of the permeate water decreases due to the changes in flux (volumetric and/or mass), permeability, pressure, and particle rejection [7,8,9,10].

PES is chosen due to its better performance and relatively low cost. The advantages of PES-based membranes manufactured are their usability in wide-range of pH values (1–12) and their good chemical resistance. On the other hand, the most significant disadvantage of PES membranes is that their resistance to clogging is low because of their hydrophobic nature. However, it is possible to increase the resistance against clogging by using PES as the matrix material of nanocomposite membranes [11,12,13,14]. PVDF membranes show hydrophobic properties. However, they are chosen due to their greater stiffness and mechanical strength.

Among other methods reported in the literature that improve the mechanical properties of polymeric membranes, the addition of inorganic particles into the polymeric matrix provides an easy, effective, and economical solution which also increases the permeability of the membranes [15,16,17,18]. The modulus of elasticity and tensile strength of polymeric materials can significantly increase even with the addition of very little amount of inorganic reinforcement [19,20]. In these studies, nanomaterials such as (GO) [21], zinc oxide (ZnO) [22], titanium dioxide (TiO_2_) [23], aluminium oxide (Al_2_O_3_) [24], carbon nanotube (CNT) [25,26], HNT [27,28,29,30,31,32,33,34,35,36,37], and SiO_2_ [38,39,40,41,42,43,44] are used to improve the mechanical strength of polymer-based membranes as well as the other functional properties of the membranes. Mohamed et al. report that by adding 0.2% HNT in weight to the polymer membrane matrix, the elasticity modulus of the membranes increases by 15.6% compared to its pure form, and the addition of more HNT causes a decrease in the modulus of elasticity [28]. In a study conducted by Khunova et al., they report that the modulus of elasticity of the polypropylene (PP) matrix increases with an addition of 5% HNT [35]. In their study, Muhamad et al. report that an addition of 2% by weight SiO_2_ to the PES membrane increases tensile strength and elongation at break [44].

On the other hand, performing experiments during the preparation of nanocomposite materials is a time and resource-consuming process. In this context, numerical simulation methods play an important role in the development of reliable nanocomposite materials [20,45,46]. Therefore, it is necessary to investigate the mechanical properties of the membranes under similar conditions to their operating environment [7]. The addition of PVP is also considered as it affects the membrane characteristics [47]. Tufekci et al. have investigated the mechanical behaviour of PVP-added polyetherimide (PEI) membranes under uniform pressure using the finite element method, and they reported that the most mechanically favourable membrane is elliptical [48]. In their next study, Tufekci et al. found that the von Mises stress decreased and the mechanical performance increased with the increase in the aspect ratio of the PVP-doped PAN elliptical membranes in the elastic zone under uniform pressure [49].

In this study, pure PES and PVDF membranes are manufactured using the phase inversion method. Production is carried out using two different membrane casting thicknesses (130 and 150 μm) in order to determine the effect of the membrane casting thickness on the characterisation parameters. Using the material properties of the polymers and the characteristics of the nanomaterials, the moduli of elasticity of nanocomposite membranes containing HNT and SiO_2_ are calculated using the Mori–Tanaka homogenisation method. Pure PES and PVDF membranes and the ones containing 1% HNT and nano SiO_2_ by weight are modelled using the finite element method. The effects of geometric shape and aspect ratio on the mechanical behaviour of the membrane are investigated by using equivalent stress (von Mises), displacement, and equivalent strain values. Each geometrical shape and material combination is modelled and compared to each other using these three criteria. This study aims to determine the membrane with the best mechanical performance based on evaluations regarding equivalent stress (von Mises), displacement, and in-plane principal strain.

## 2. Materials and Methods

### 2.1. Material

**Figure 1:**
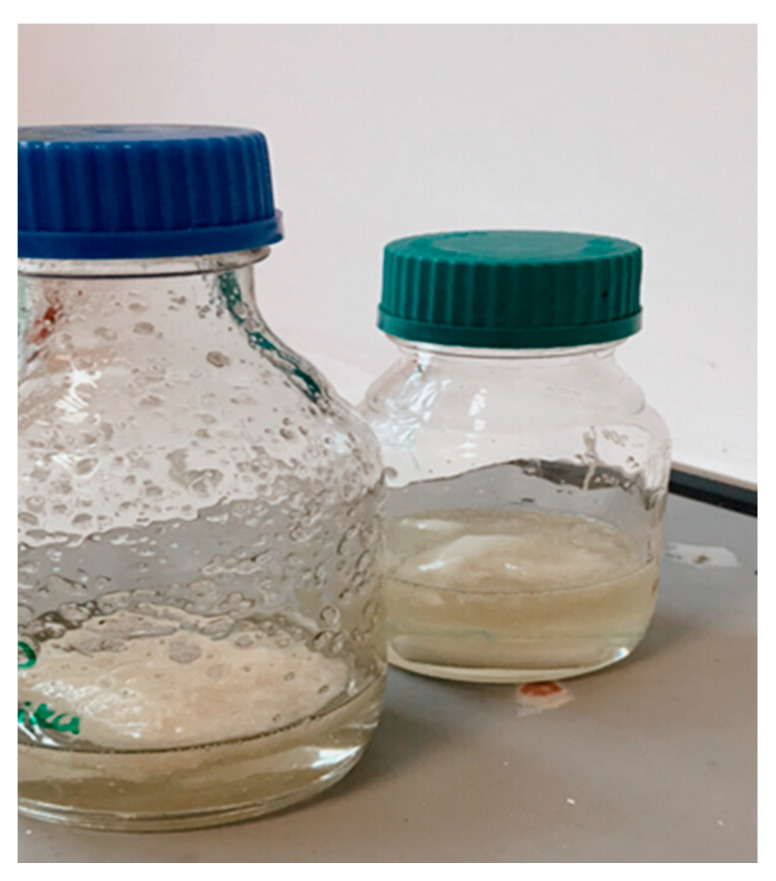
PES and PVDF membrane solutions.

PES (Mw = 62–64 kDa) and PVDF (Mw = 300–320 kDa) polymers used in this study are obtained from Solvay. PVP (Mw = 10 kDa) is acquired from Sigma-Aldrich (St. Louis, MO, USA) and N-Methyl-2-pyrrolidine (NMP) from Ashland. PES and PVDF membrane solutions can be seen in Figure 1.

### 2.2. Membrane Synthesis

**Figure 2:**
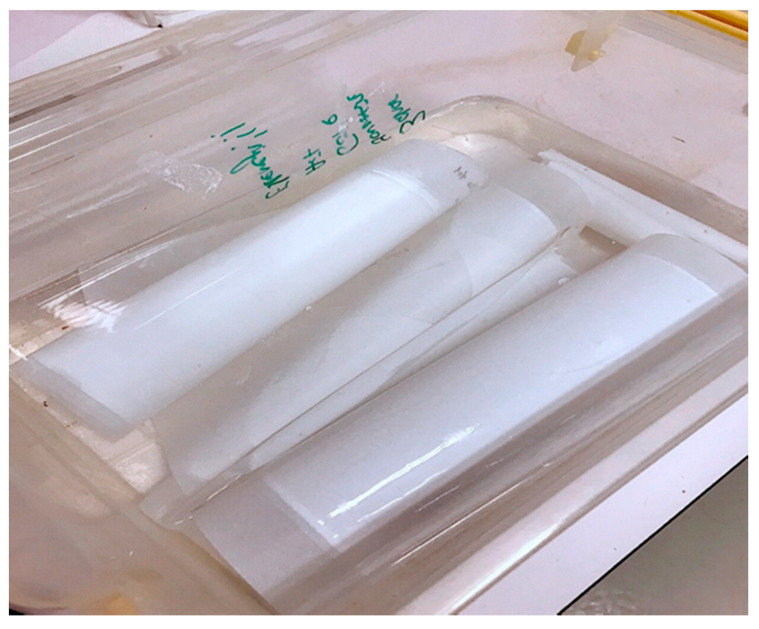
Manufactured membranes.

To synthesise the membranes, 8% PVP is added into 76% NMP by weight to form pores in the membrane and they are mixed at 60 °C and 200 rpm until the PVP is completely dissolved. Then, 16% of PES or PVDF is added. The solutions containing PES are mixed at 60 °C and 200 rpm and the solutions containing PVDF are mixed at 70 °C and 200 rpm for 1 day until they are fully homogenised. The castings of polymer solutions are brought to room temperature and prepared using automatic film applicator (Sheen) at a speed of 80 mm/s. The casting of PES and PVDF solutions is conducted on a support non-woven layer made of polyethylene terephthalate (PET) in two different thicknesses of 130 and 150 μm and on a glass plate (without a support layer) with a thickness of 130 μm. The cast membranes are quickly immersed in a water bath containing ultrapure water. Synthesised membranes are washed thoroughly with ultrapure water and then kept in ultrapure water at +4 °C until they are used in the characterisation studies. The manufactured membranes are shown in Figure 2.

### 2.3. Membrane Characterisation

#### 2.3.1. Polymer Solution Viscosity

Before membrane casting, the viscosity measurement of the polymer solutions at room temperature is performed using a viscometer (AND SV-10).

#### 2.3.2. Membrane Surface Characterisation

In this study, FTIR is performed to determine the presence of functional groups belonging to the polymer and the PVP used in the synthesised polymeric membranes which ensured that the manufactured membranes consisted of the same functional groups and no chemical compound could influence the mechanics of the membrane material.

FTIR analysis is performed using the FTIR spectrometer (Perkin Elmer, Inc., Waltham, MA, USA) to determine the functional groups on the surface of the membranes and spectra are obtained in the 4000–650 cm^−1^ wavenumber range. In order to characterise the surface morphology of the membranes, the insulating polymeric membranes are first coated with Gold-Palladium (Au-Pd) to make them conductive.

This is followed by the acquisition of the SEM images of the membranes by magnifying 10,000 times at 20 kV with a scanning electron microscope (SEM, FEI, Quanta Feg 250).

#### 2.3.3. Hydrophilicity and The Minimum Pore Size

A contact angle measuring device, KSV Attension Theta (Biolin Scientific, Västra Frölunda, Sweden) is used to determine the hydrophilicity of the membranes. The contact angles of the membranes are identified by dropping pure water on the membrane surfaces at room temperature. The average value is taken by obtaining 5 measurements for each membrane.

In order to determine the water content of the membranes, they are kept in ultra-pure water for 24 h. Immediately after the membranes are removed from the ultrapure water, the moisture on the surface of the membranes is quickly removed with the drying paper and the membranes are weighed while they are wet. Afterwards, the membranes are dry weighed following 48 h in the oven at 45 °C. The water content of the membranes is calculated using Equation (1):

(1) $$\mathrm{Water}\;\mathrm{content}\;(\%)=\frac{m_{\mathrm{wet}-}m_{\mathrm{dry}}}{m_{\mathrm{wet}}}\times 100$$

The minimum pore diameter of the membranes is determined with a porometer Quantachrome 3G Porometer (Anton Paar Instruments, Graz, Austuria).

#### 2.3.4. Membrane Pure Water Permeability Test

**Figure 3:**
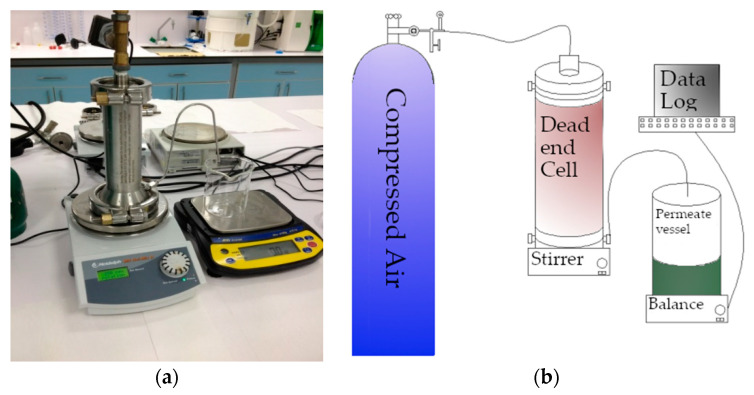
View of permeability test (**a**) experimental set-up (**b**) schematic diagram.

The permeability test is performed with a continuously stirred dead-end filtration cell, Sterlitech, HP4750 (Sigma Aldrich, St. Louis, MO, USA), pressurised by nitrogen gas with a capacity of 300 mL and an active membrane area of 14.6 cm^2^. The experimental set-up (Figure 3a) and its schematic view (Figure 3b) are shown in Figure 3. In dead-end filtration, the water flows perpendicular to the membrane surface. Here, all the water passes through in the dead-end cell as the permeate, and there is no rejection of water. Permeate flux is obtained by measuring the weight of the permeate. Meanwhile, the dead-end filtration system is stirred at a constant speed of 200 rpm at room temperature.

First, the compression process is applied to the synthesised membranes at 5 bar pressure for 30 min until their flux values stabilised. After the compression process, pure water filtration is carried out for 11 min at three different pressure values (1, 1.5, and 2 bar). The experiments are repeated three times for each membrane at all pressure values. The average flux value of each membrane is calculated using Equation (2).

(2) $$J=\frac{V}{A∆t}$$

Here, J indicates flux (L/m^2^.h), V indicates the permeate volume (L), A indicates the membrane area (m^2^), and Δt (h) indicates the filtration time.

The membrane permeability is defined as the volume flowing through the membrane per unit area, time, and pressure (ΔP). The permeability value of the membranes is determined from the slope of the line fitted by the linear regression method in the flux-pressure graphs or from the data by using Equation (3) where R is the permeability.

(3) $$R=\frac{J}{∆P}$$

#### 2.3.5. Dynamic Mechanical Analysis (DMA)

The membranes made of pure PES and PVDF flat sheet of 130 µm thickness without nano-reinforcements are put through the tensile testing process at room temperature using a dynamic mechanical analyser DMA, SII Exstar DMS 6100 (Seiko Instruments Inc., Chiba, Japan) to determine the mechanical properties of the membranes without a support layer.

The experiment is carried out by using an electric motor that had the capability to apply dynamic and different sorts of loadings. When the experiment is completed, in other words, when the membranes failed, the tensile strength values of the membranes are calculated from the stress-strain graphs and the moduli of elasticity of the membranes are calculated from the slope of the line in the linear elastic deformation region of the graphs.

### 2.4. Numerical Modelling

The purpose of building these numerical models for this study is to generate a self-consistent environment to investigate the effects of changing parameters such as the addition of nano-sized reinforcements and the geometry of the membranes on the mechanical behaviour of the membrane systems. This environment lets one compare the outcomes of those models without having to conduct actual experiments.

#### 2.4.1. Calculation of Elasticity Modulus of Nanocomposite Membranes by Mori–Tanaka Homogenisation Method

The composite materials are assumed to be homogeneous in order to facilitate further calculations and analysis. The calculations of the modulus of elasticity of HNT or nano SiO_2_ doped PES and PVDF membranes are performed by the Mori–Tanaka homogenisation method. Here, the matrix is assumed to be an elastoplastic material whereas the reinforcements are assumed to be perfectly elastic. This assumption is based on the fact that the matrix fails long before the reinforcements approach their limits of the linear elastic regime due to the significant differences in their stiffnesses.

Each of these materials is also assumed to be isotropic and homogeneous. The SiO_2_ particles are considered to be perfect spheres while the HNT particles are considered to be ellipsoids. Furthermore, for the calculations, the density values of PES and PVDF polymers and the results obtained from the tensile test applied to pure PES and PVDF membranes are used as well as the density, the modulus of elasticity, the average dimensions of HNT, and nano SiO_2_. The values for the membrane matrix and the nanomaterials used in the computations are given in Table 1. The numerical values of the properties of HNT and nano SiO_2_ given in Table 1 are taken relying on the information in the literature [50,51].

The nanocomposite membranes containing 16% PES or PVDF, 8% PVP, 75% NMP, and 1% nanomaterials (HNT or nano SiO_2_) by weight are homogenised within the scope of this study. It is also among the considerations that HNT and nano SiO_2_ have a random distribution in the structure and the nanocomposite materials are therefore assumed to be quasi-isotropic.

#### 2.4.2. Finite Element Modelling of Membranes

**Figure 4:**
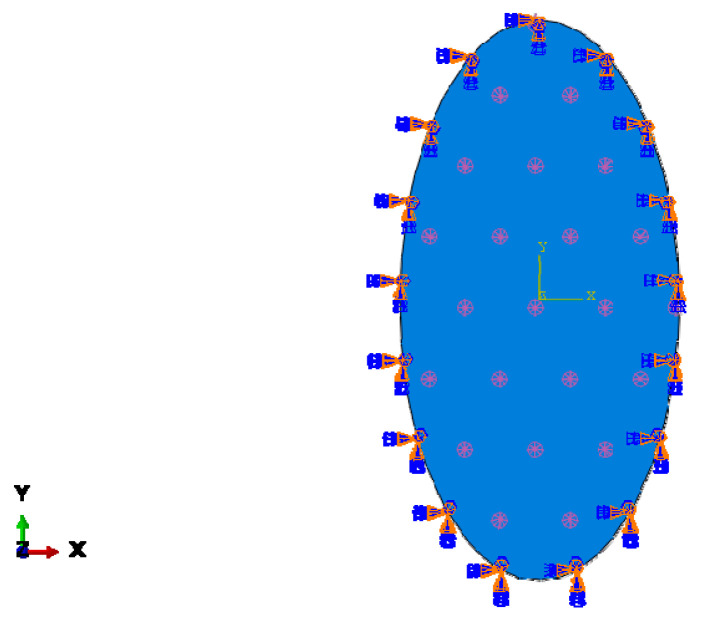
Boundary conditions and loads in the finite element model.

To determine the effect of the membrane shape and the aspect ratio on the mechanical behaviour of the membranes, they are modelled employing the finite element method. For modelling, first the mechanical properties of the polymeric membranes are extracted from the stress-strain curves obtained by the tensile tests. These properties are used in both the Mori–Tanaka homogenisation of the nanocomposite membranes and the finite element modelling of all membranes. Membranes are modelled statically with finite element models considering the geometric and material nonlinearities. The material nonlinearities stemmed from the elastoplastic behaviour of the polymeric membrane materials which are also assumed to be isotropic and homogenous. For the numerical analysis, various geometries are generated for pure and nanocomposite membranes in order to determine the effect of the geometric form on the mechanical behaviour with the same surface area, under the same loads, and boundary conditions. A total of 15 geometric shapes are considered for this study for each membrane material, namely, hexagonal, circular, square, and ellipses with 12 different aspect ratios from 1.25 to 4. The pressure applied to the membranes and the surface area of the membranes is the same for all the geometric shapes in order to ensure that the total load is equal on each membrane surface. The membranes are fixed from their outer edges and a uniform pressure of 3500 N/m^2^ is applied to the surfaces of the membranes. These boundary conditions and the loads are believed to represent the actual operational environment. The boundary conditions and the loads are visualised in Figure 4. For the discretisation, six-node triangular shell elements are defined with second-order shape functions. Each node had six degrees of freedom. The results of these simulations are compared in terms of three different criteria: equivalent stress (von Mises), displacement, and in-plane principal strain.

## 3. Results and Discussion

### 3.1. Membrane Characterisation Results

#### 3.1.1. Viscosity of Membrane Casting Solutions

The viscosity of the membrane casting solution affects the membrane morphology as it plays a role in the rate of transition between solvent/non-solvent in phase inversion [52]. The viscosities of the casting solutions containing PES or PVDF are measured at 25 °C before the membrane casting and are found as 9.55 Pa s and 12 Pa s, respectively. Greater viscosity is usually requires to prevent excessive solution penetration into porous support materials for flat sheet membranes [53]. Due to the higher average molecular weight of PVDF, the higher viscosity of the membrane casting solution containing PVDF is an expected result.

#### 3.1.2. FTIR and SEM

**Figure 5:**
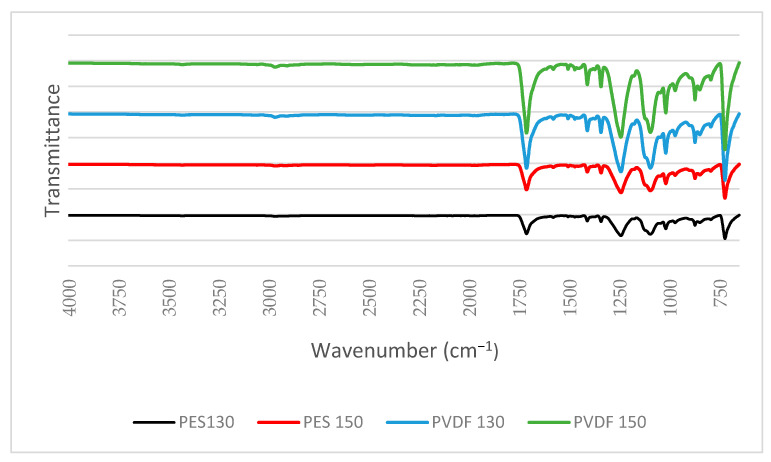
FTIR spectra of PES and PVDF membranes.

In the spectra obtained from the results of the FTIR analyses, shown in Figure 5, the peaks seen at 1409 cm^−1^ and 1340 cm^−1^ in PES membranes correspond to the S=O stress vibrations, and the peaks seen at 1018 cm^−1^ and 2971 cm^−1^ in PVDF membranes correspond to the stress and CH_2_ stretching vibrations, respectively [54,55]. The peaks seen at 1713 cm^−1^ in the spectra occur due to the C=O stretching vibration of the carbonyl group originating from PVP [56,57].

**Figure 6:**
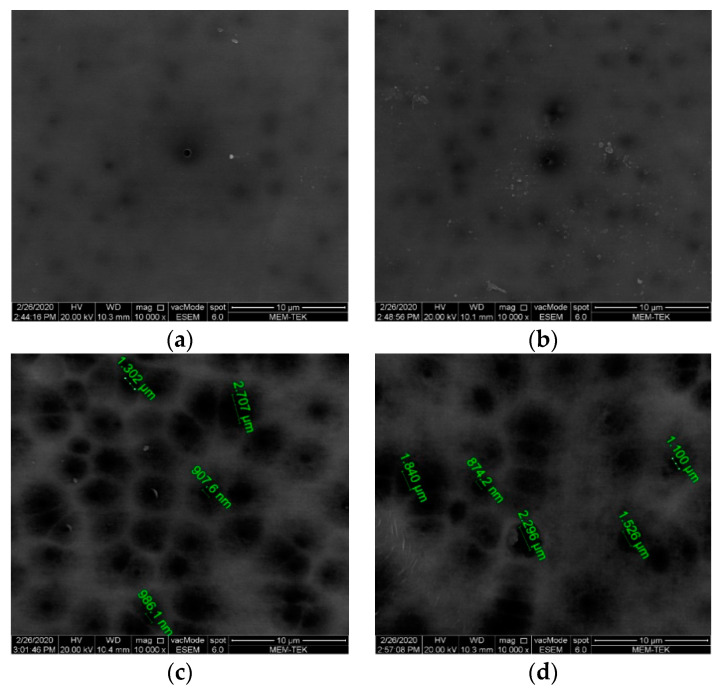
SEM surface images of membranes (**a**) PES 130, (**b**) PES 150, (**c**) PVDF 130, and (**d**) PVDF 150.

From the SEM surface images of the membranes presented in Figure 6, it is seen that the PES membranes have a relatively dense surface compared to the PVDF membranes. In addition to more pore formation on the surfaces of PVDF membranes, larger pores are formed. In the phase inversion process, fewer macro-pores, which usually initiate on the surface where the least polymer content is, are formed, if there is a smaller distance between the lower and upper surfaces of the membrane, in other words, if the thickness of the casting solution is lower [58].

#### 3.1.3. Surface Hydrophilicity and Water Content of Membrane

**Figure 7:**
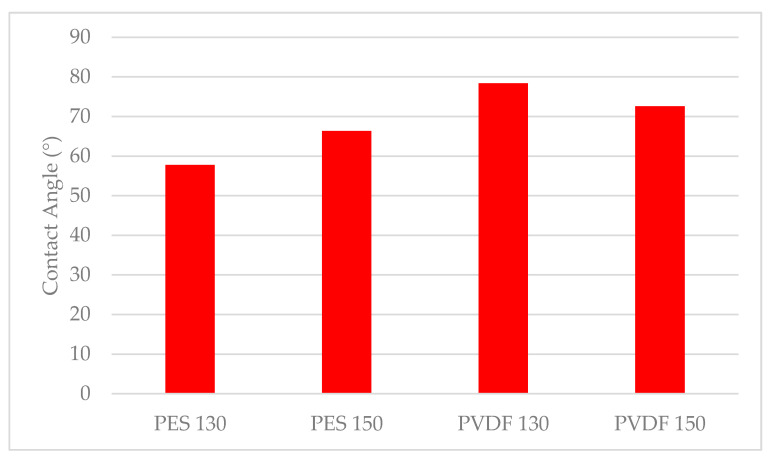
Membrane surface contact angles in degrees.

It is determined that all the synthesised membranes have hydrophilic properties since their contact angle is less than 90°. It is also determined that PES membranes have lower contact angle values compared to PVDF membranes, in other words, they are more hydrophilic (Figure 7). In other studies, while the contact angle value of PVDF membranes without PVP is found to be greater than 90°, [54,59,60] the contact angle of PVP-added PVDF membranes is found to be lower than 90°, similar to the results obtained in this study [61]. From the contact angle values obtained for PVP-doped PES membranes in the literature, it is observed that hydrophilic membranes are produced in accordance with the contact angle values obtained in this study [56,62,63]. As the membrane casting thickness increases, the contact angle of PES membranes increases while the contact angle of PVDF membranes decreases. Since the contact angle is a parameter related to the membrane surface, it is considered that there is no clear relationship between casting thickness and contact angle.

**Figure 8:**
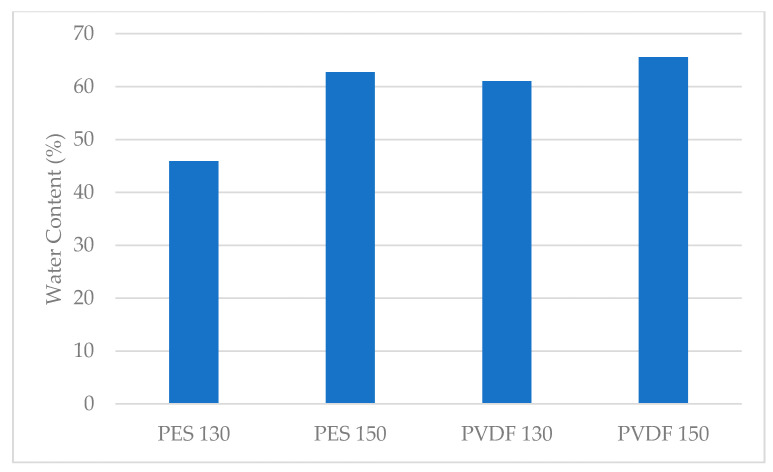
The water content of membranes.

Figure 8 displays the results of the water content measurements of the membranes calculated using Equation (1) as mentioned above. Comparing the synthesised membranes, it can be stated that the membrane with the highest water content is 65.57% and PVDF 150. It is determined that the least water-containing membrane is PES 130 with 45.93%. It can also be seen that as the thickness increases, the water content of the PES and PVDF membranes also increases. The pore size is correlated to the water content and depends on the thickness of the membrane casting solution. As the thickness of the casting solution increases, the macro-scale pore formation in the membrane also increases [58]. The formation of larger-sized pores on the membrane surface causes an increase in water retention within the pores [64].

#### 3.1.4. Membrane Pore Size

As can be seen from the minimum pore diameter results of the membranes given in Table 2, all the synthesised membranes are at the microfiltration level [2]. Moreover, the SEM surface images conform to the minimum pore diameter results, validating the fact that the manufactured membranes are microfiltration membranes. Although the minimum pore diameters are very similar in all the synthesised membranes, the maximum value of the minimum pore diameter is detected in the PVDF 150 membrane with 0.207 µm. As the thickness increases in both the PES and PVDF membranes, the minimum pore diameter also increases.

#### 3.1.5. Pure Water Permeability Performance of Membranes

**Figure 9:**
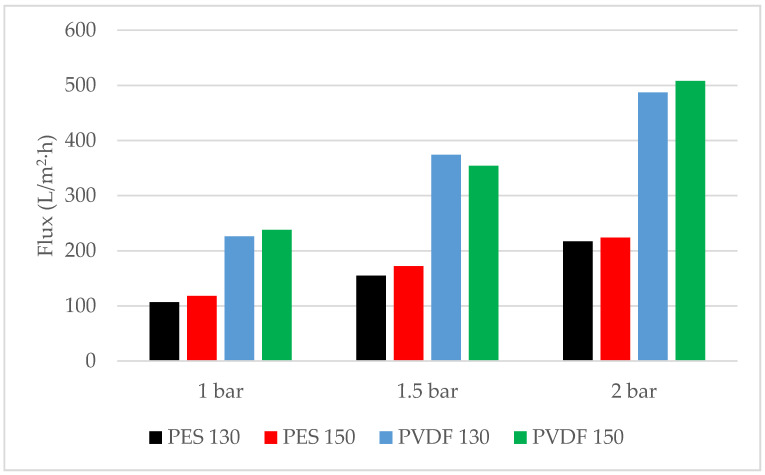
Flux values of membranes at different pressures.

**Figure 10:**
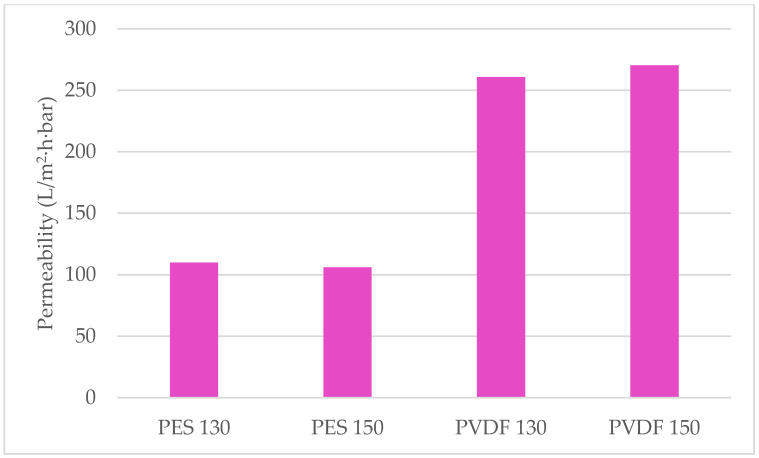
Permeability of membranes.

From the flux values of the membranes shown in Figure 9 at three different pressures, the highest flux is 507.89 L/m^2^·h at 2 bar pressure on a PVDF 150 membrane whereas the lowest flux is reached at 1 bar pressure, 107.06 L/m^2^·h, on a PES 130 membrane. Figure 10 shows that the highest permeability is reached in a PVDF 150 membrane with 270.38 L/m^2^·h·bar. The permeability of PVDF membranes is found to be approximately 2.5 times higher compared to PES membranes. It is thought that this is caused by the formation of more pores in the PVDF membranes compared to PES membranes, as can also be seen in the SEM images (Figure 6), and the decrease in the hydraulic resistance of PVDF membranes due to the larger pores formed.

Unlike the results reported in the literature, an increase in the permeability of the membranes could not be observed with decreasing contact angle in the results of this study [25,59]. However, in this study, this correlation cannot be found. The measurement of contact angles are dominantly affected by the pore structure of the membrane surface whereas the pure water permeability is related to the pores in the internal structure as well as the pores on the surface of the membrane. The pores in the internal structure of PVDF membranes form in a way that allows water to pass more easily which dominates the overall permeability of the membranes.

### 3.2. Mechanics of the Membranes and Their Materials

**Figure 11:**
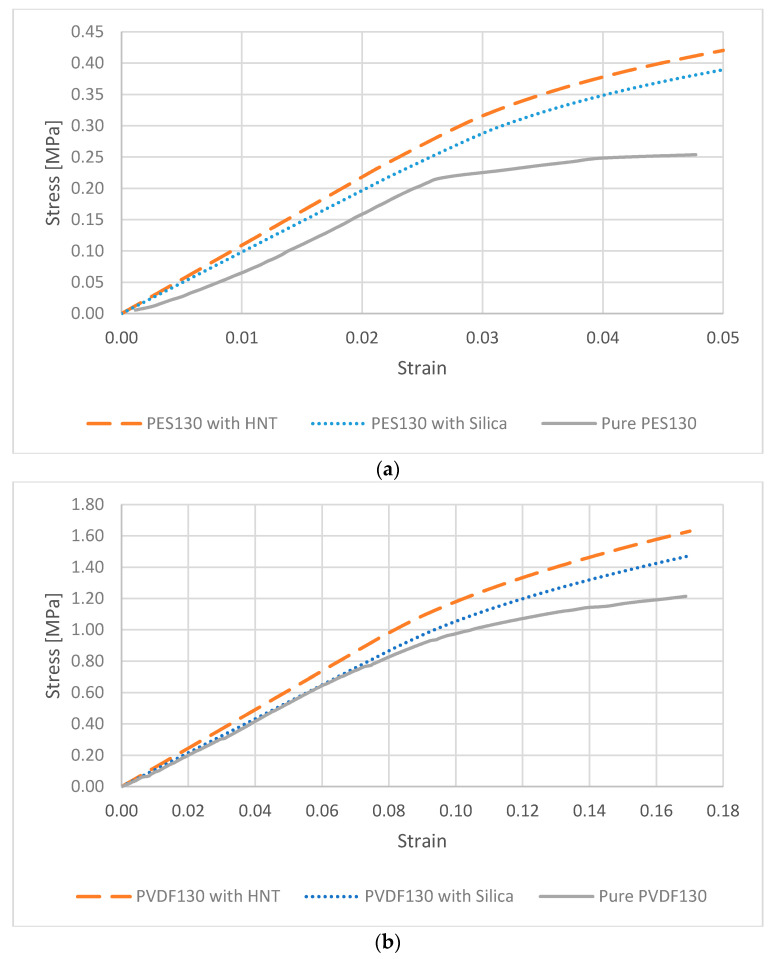
Stress-strain curves for the membranes (**a**) PES130 and its nanocomposites (**b**) PVDF130 and its nanocomposites.

The stress-strain graphs obtained from the experimental and modelling processes applied to pure PES and PVDF membranes and their nanocomposites with 1% HNT and nano-SiO_2_ by weight are shown in Figure 11.

The moduli of elasticity for the 130 µm thick PES and PVDF membranes are determined as 8.7 MPa and 10.5 MPa, respectively, through the tensile tests. It is important to note that there are no support layers attached to the membrane samples. The PVDF membrane has a greater modulus of elasticity compared to the PES membrane and this situation indicates that the membrane is more rigid and less deformed. The tensile strength of the PES membrane is 0.254 N/mm^2^, while the PVDF membrane is 1.214 N/mm^2^ (approximately 4.8 times greater). The results obtained from the mechanical tests show that the mechanical properties are significantly affected by factors such as the molecular weight of the polymer, the porosity, and consequently the PVP used in membrane production. Depending on these, there are various results that differ considerably from each other that are available in the literature [16].

**Figure 12:**
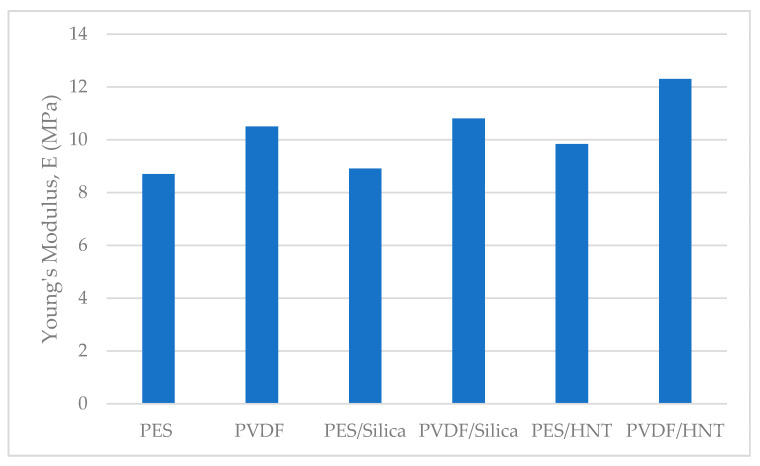
Young’s modulus of pure and nanocomposite membranes.

The homogenization process yields the moduli of elasticity for PVDF/HNT, PVDF/SiO_2_, PES/HNT, and PES/SiO_2_ nanocomposite membranes as 12.3 MPa, 10.8 MPa, 9.84 MPa, and 8.91 MPa, respectively. These results are presented in Figure 12. With the addition of SiO_2_ to PES and PVDF membranes, the modulus of elasticity increased by 2.4% and 2.8%, respectively, while with the addition of HNT, the moduli of elasticity increased by 13% and 17%, respectively. The modulus of elasticity of the pure PVDF membrane without any nanomaterial addition is found to be higher than the of elasticity of pure PES, PES/HNT, and PES/SiO_2_ membranes. Even though the same mass fractions of the reinforcements of HNT and SiO_2_ are added to the polymers, it is observed that the modulus of elasticity of nanocomposites reinforced with HNT increased more than the ones reinforced with SiO_2_. This can be explained with the greater modulus of elasticity (140 GPa) than the modulus of elasticity (70 GPa) of SiO_2_, and the thin and long tubular structure of HNT which may significantly hinder the deformations of the matrix that are not in the same direction as the orientation of the tubes. Considering that the orientations of the HNTs are random, a quasi-isotropic and stiffer behaviour is a predictable outcome.

### 3.3. Numerical Analysis of Pure and Nanocomposite Membranes by Finite Element Method

The results of the numerical analysis, given in Table 3, express the maximum value appearing in the membrane for each criterion, namely, equivalent stress, displacement, and in-plane principal strain of pure PES and PVDF membrane in different geometries.

Among all PES membranes with different geometric shapes, the most favourable membrane in terms of mechanical performance is the elliptical PES membrane with an aspect ratio of 4 due to the lower equivalent stress and displacement values. The weakest PES membrane in terms of mechanical behaviour is the square membrane due to the higher equivalent stress values compared to other geometries. Equivalent stress and displacement values are obtained as 0.4364 MPa, 2.546 mm for the elliptical PES membrane with an aspect ratio of 4, respectively. The highest equivalent stress value is observed in the square PES membrane as 0.5597 MPa.

In the PVDF membrane, the highest equivalent stress value (0.5983 MPa) is detected on the square membrane. The lowest values of equivalent stress and displacement are detected on the elliptical PVDF membrane with an aspect ratio of 4 as 4.664 MPa and 2.347 mm. It can be said that an elliptical membrane with an aspect ratio of 4 is the most suitable and the square membrane is the most unsuitable for usage.

In both pure polymeric membranes, comparing the hexagonal, square, and circular membranes, the membrane with the most desirable shape is the circular membrane, whereas the membrane with the most undesirable geometry is determined as the square membrane. Even though the circle deforms more than the other three shapes, considering that the lowest equivalent stress value is observed in the circular membrane, it is expected to withstand greater loads.

With increasing aspect ratios in elliptical PES and PVDF membranes, the equivalent stress values increased until an aspect ratio of 1.75 and the in-plane principal strain values increased until an aspect ratio of 3.75 and then they decreased. Additionally, displacement values decreased with the aspect ratio increment. This situation indicates that the ellipse membrane with an aspect ratio of 4 selected for PES and PVDF can withstand higher pressure than the other elliptical membranes and it can be said that these elliptical membranes with aspect ratios of 4 are the ones that can be expected to carry the greatest loads among the presented geometries.

**Figure 13:**
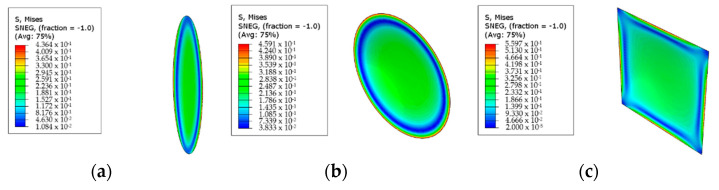
Visualised equivalent stress results of PES membranes: (**a**) ellipse 4 (**b**) circle (**c**) square membrane.

Ellipse membranes with an aspect ratio of 4 represent the better membranes in terms of mechanical performance, not only because the maximum numerical values of all three criteria are lower than other geometries but also because the stress is distributed more evenly compared to the other membranes. This can be seen in Figure 13 which displays elliptical with an aspect ratio of 4 (a), circular (b) and square (c) PES membranes. The concentration of greater values of stress in a narrow region around the centre of the membrane does not represent the desired stress distribution, as it will accelerate the local failure. It is determined that as the aspect ratio increases, the maximum of the numerical values decreases. The distribution also becomes more even, and the membrane can be expected to display a better mechanical performance.

**Figure 14:**
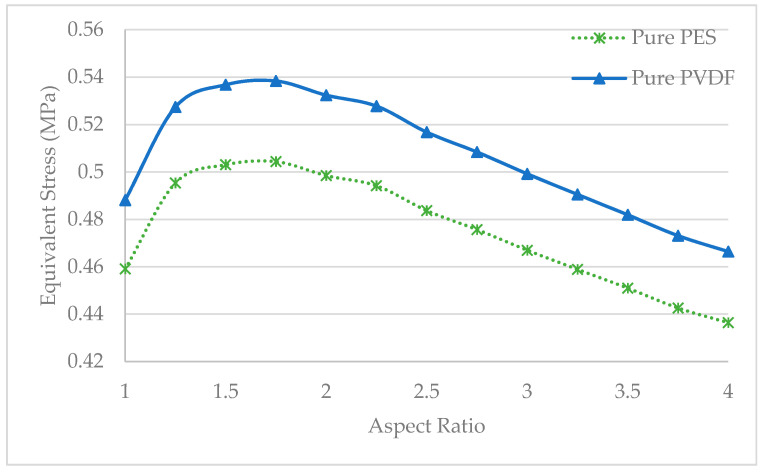
Maximum equivalent stresses of pure membranes of different aspect ratios.

In Figure 14 the equivalent stress values of each pure membrane with aspect ratios from 1 to 4 are displayed. There it can be seen that the pure PVDF membrane showed a higher equivalent stress value than the pure PES membrane in all geometries. This occurs due to the greater modulus of elasticity of PVDF compared to PES.

**Figure 15:**
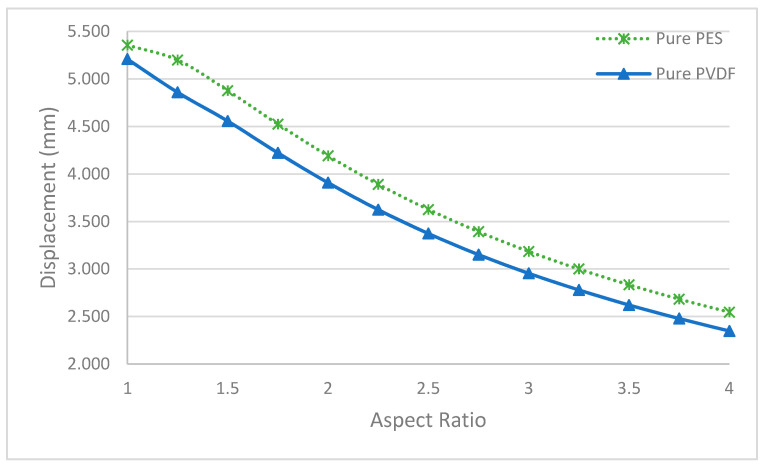
Maximum displacements of pure membranes with different aspect ratios.

In Figure 15, it is shown that there is an inverse correlation between the aspect ratio and the maximum displacement value. Hence, the lowest maximum displacement value is obtained in elliptical membranes with the highest aspect ratio (a/b = 4). Because of the stiffer nature of the PVDF membrane, fewer displacement values are observed.

**Figure 16:**
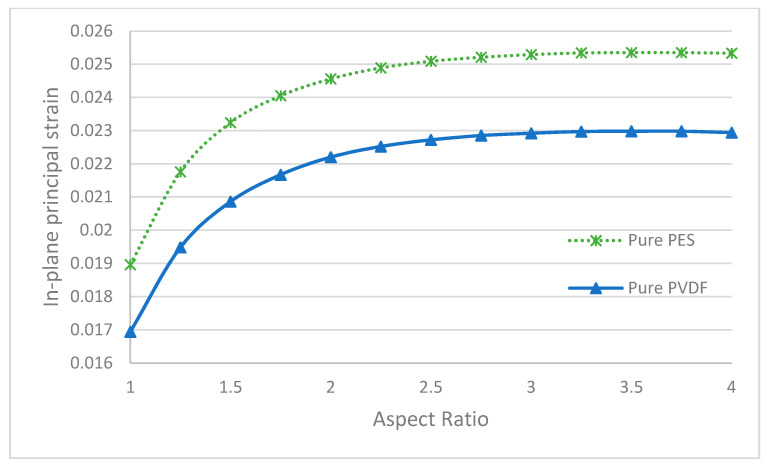
The maximum in-plane principal strain of pure membranes with different aspect ratios.

In Figure 16, the maximum in-plane principal strain values of pure membranes with different aspect ratios are given. It can be claimed that the in-plane principal strain values of pure PVDF membranes are less than the pure PES membranes, similar to the displacement results. Even though the displacement value fell with increasing aspect ratios, the strain values rose.

Table 4 and Table 5 present the results of the nanocomposite membranes in all geometric forms considered within the scope of this study. Among all nanocomposite membranes with different geometric shapes, the lowest equivalent stress value is observed in the PES/SiO_2_ ellipse membrane with an aspect ratio of 4 and the lowest displacement value is observed in the PVDF/HNT ellipse membrane with an aspect ratio of 4 (Table 4 and Table 5). The highest equivalent stress value is observed in the PVDF/HNT square membrane.

Displacement values and in-plane principal strain values differed for each geometric shape in each nanocomposite membrane. Among all the nanocomposite membranes, elliptical membranes with aspect ratios of 4 showed the best mechanical performance. Moreover, square membranes can be noted as the weakest in terms of mechanical performance.

Visualising the numerical results in all the nanocomposite membrane combinations, it is seen that the distributions of equivalent stress, displacement, and in-plane principal strains have similar characteristics. Among all the geometric shapes in nanocomposite membranes, the highest values of equivalent stress are at the edges of the membranes; the lowest values are concentrated in the centre of the membranes. It is determined that the regions with lower equivalent stresses concentrated in the centre of the ellipse membranes expand towards the endpoints of the ellipse as the aspect ratio increases, whereas the region with the equivalent stress occurring on the long sides decreases.

It is important to emphasise that the greatest value of the maximum equivalent stress in membranes is obtained in the square membrane and the lowest value is obtained in the elliptical membrane with an aspect ratio of 4. These results show that elliptical nanocomposite membranes with an aspect ratio of 4 can withstand higher loads than other elliptical membrane geometries and, therefore, this geometrical shape is found to be the most suitable in terms of mechanical performance.

**Figure 17:**
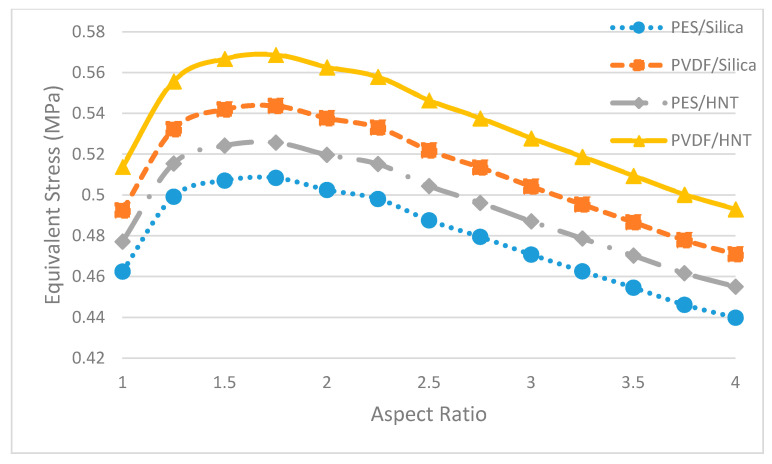
Maximum equivalent stresses of nanocomposite membranes of different aspect ratios.

Figure 17 shows the equivalent stress values of each nanocomposite membrane with aspect ratios from 1 to 4. This graph shows that the PVDF/HNT nanocomposite membranes display the highest equivalent stresses. This happens due to the stiffer nature of this PVDF/HNT nanocomposite. In addition, the PES/SiO_2_ nanocomposite membrane showed the lowest equivalent stress values of all the geometries. This is the consequence of the compliance of the PES/SiO_2_ nanocomposite.

**Figure 18:**
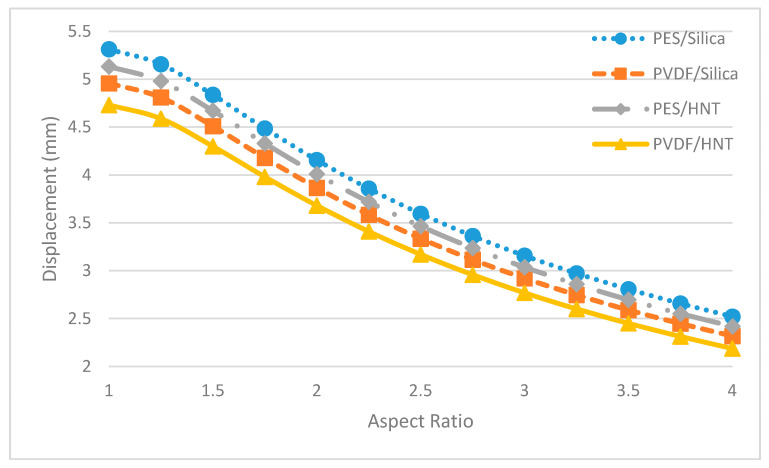
Maximum displacements of nanocomposite membranes with different aspect ratios.

In Figure 18, it is seen that a decrease in maximum displacements occurs in all of the nanocomposite membranes with an increasing aspect ratio. In the numerical analysis, the least maximum displacement is observed in the elliptical membranes with the highest aspect ratios (a/b = 4). Among all the elliptical membranes, it is determined that the membrane with the least displacement for all aspect ratios examined is the PVDF/HNT membrane. Again, the lesser displacements are expected as the PVDF/HNT nanocomposite is stiffer compared to the rest of the material groups considered in this study.

**Figure 19:**
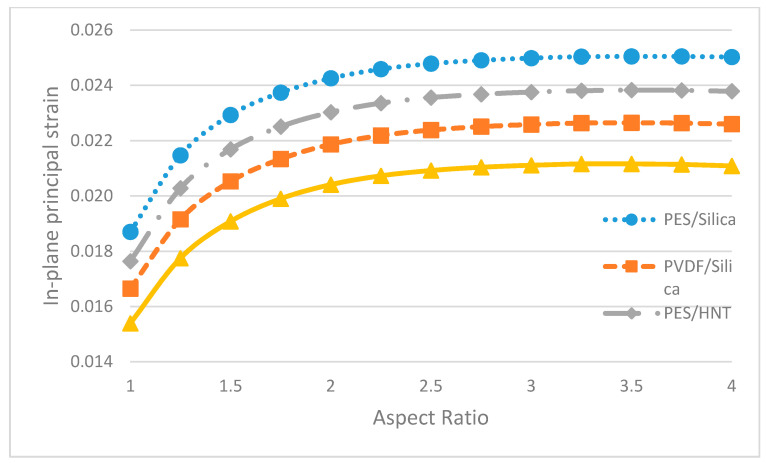
The maximum in-plane principal strain of nanocomposite membranes with different aspect ratios.

In Figure 19, the maximum in-plane principal strain values of nanocomposite membranes with different aspect ratios are given and from Figure 19 it can be said that the in-plane principal strain values of HNT-reinforced membranes are found to be less than their SiO_2_ doped, similar to the displacement results. However, the displacement results show a falling trend with increasing aspect ratios, whereas the strain values tend to rise with increasing aspect ratios.

Finally, it is important to highlight that geometric shape is a dominant factor affecting the mechanical performance of a membrane. The stress, strain and displacement criteria show very similar trends for each material with respect to geometry. The presence of sharp corners, such as in squares, cause significantly greater stresses whereas the circular membrane, which shows lower stress levels, has no sharp corners. The hexagonal membrane has characteristics that lie in-between the square and circular membranes. This phenomenon shows that, for the same aspect ratio, smoother geometries have a superior mechanical performance over the geometries with sharp corners. From a treatment point of view, it is known that dead zones emerge on the membrane geometries with sharp corners, for example in rectangular or square membranes. In hexagonal membranes, those dead zones are expected to be relatively smaller and in circular membranes, they are either expected to be significantly smaller or not expected at all.

Moreover, on the geometry of the membranes, it is also observed that the aspect ratio is another dominant factor that has an impact on the mechanical performance of the membranes. This is shown with the elliptical membranes that have no sharp corners to avoid any possible effects of stress concentrations. Moreover, it is predicted that the dead zones that emerge would be smaller in elliptical membranes than in rectangular membranes. Hence, the overall performance of elliptical membranes would be better both mechanical- and treatment-wise.

## 4. Conclusions

In this study, synthesised and characterised PES and PVDF-based microfiltration membranes are modelled by numerical analysis to predict their mechanical performance. As a part of the characterisation process, the pure water permeability values are measured and it is determined that the permeability of pure PVDF membranes is approximately 2.5 times higher than PES membranes. Using the modulus of elasticity determined by applying tensile tests to the synthesised pure membranes, the moduli of elasticity of the PES and PVDF nanocomposite membranes with 1% HNT or nano SiO_2_ by weight are determined approximately by the Mori–Tanaka homogenisation method without any mechanical testing. With 1% HNT or nano SiO_2_ addition, it is determined that the modulus of elasticity of the nanocomposite PES and PVDF membranes increased and the membranes became stiffer compared to their pure forms. In pure PES and PVDF membranes modelled by numerical analysis with the finite element method, elliptical membranes with aspect ratios of 4 are found to be the most suitable shape among the ones investigated within the scope of this study in terms of mechanical performance. For nanocomposite PES and PVDF membranes, it is determined that elliptical membranes with an aspect ratio of 4 can perform better than the others considered within this study. After all the membranes are modelled with the finite element method, the best membrane in terms of both pure water permeability performance, stiffness, and mechanical performance is the 1% HNT added PVDF elliptical membrane with an aspect ratio of 4.

This study reveals that shape and aspect ratio affect the mechanical behaviour of the membranes that are used in the treatment of drinking and/or wastewater. The shape does not only mean a limitation for the design of membrane systems. Designing the membranes by selecting the most appropriate shape in terms of mechanical strength will contribute to the prolongation of its service life and reduce its cost.

## Figures and Tables

**Table 1:** Mechanical properties of materials used in homogenisation method.

	Matrix		Nanomaterial	
	PES	PVDF	HNT	Nano SiO_2_
Density (kg/m^3^)	1370	1800	2365	1200
Young’s modulus (MPa)	8.7	10.5	1.4 × 10^5^	7 × 10^4^
Mean diameter (nm)	−	−	50	20
Length to diameter ratio	−	−	30	1

**Table 2:** Minimum pore diameters of the membranes.

Membrane	Minimum Pore Diameter (µm)
PES 130	0.182
PES 150	0.193
PVDF 130	0.184
PVDF 150	0.207

**Table 3:** Numerical analysis results of pure PES and PVDF membranes in different geometries.

		PES			PVDF	
	Equivalent Stress (N/mm^2^)	Displacement (mm)	In-Plane Principal Strain	Equivalent Stress (N/mm^2^)	Displacement (mm)	In-Plane Principal Strain
Hexagon	5.467 × 10^−1^	5.261	2.108 × 10^−2^	5.846 × 10^−1^	4.918	1.862 × 10^−2^
Circle	4.591 × 10^−1^	5.357	1.896 × 10^−2^	4.881 × 10^−1^	5.009	1.694 x10^−2^
Square	5.597 × 10^−1^	5.011	2.290 × 10^−2^	5.983 × 10^−1^	4.681	2.030 × 10^−2^
Ellipse 1.25	4.954 × 10^−1^	5.200	2.176 × 10^−2^	5.274 × 10^−1^	4.860	1.948 × 10^−2^
Ellipse 1.5	5.031 × 10^−1^	4.878	2.324 × 10^−2^	5.368 × 10^−1^	4.557	2.086 × 10^−2^
Ellipse 1.75	5.044 × 10^−1^	4.525	2.405 × 10^−2^	5.384 × 10^−1^	4.222	2.167 × 10^−2^
Ellipse 2	4.985 × 10^−1^	4.192	2.456 × 10^−2^	5.324 × 10^−1^	3.908	2.220 × 10^−2^
Ellipse 2.25	4.942 × 10^−1^	3.892	2.489 × 10^−2^	5.278 × 10^−1^	3.624	2.252 × 10^−2^
Ellipse 2.5	4.837 × 10^−1^	3.627	2.509 × 10^−2^	5.168 × 10^−1^	3.373	2.272 × 10^−2^
Ellipse 2.75	4.757 × 10^−1^	3.393	2.521 × 10^−2^	5.084 × 10^−1^	3.151	2.285 × 10^−2^
Ellipse 3	4.670 × 10^−1^	3.185	2.529 × 10^−2^	4.992 × 10^−1^	2.954	2.292 × 10^−2^
Ellipse 3.25	4.589 × 10^−1^	3.000	2.534 × 10^−2^	4.905 × 10^−1^	2.779	2.297 × 10^−2^
Ellipse 3.5	4.510 × 10^−1^	2.834	2.535 × 10^−2^	4.819 × 10^−1^	2.620	2.298 × 10^−2^
Ellipse 3.75	4.426 × 10^−1^	2.683	2.535 × 10^−2^	4.731 × 10^−1^	2.478	2.298 × 10^−2^
Ellipse 4	4.364 × 10^−1^	2.546	2.533 × 10^−2^	4.664 × 10^−1^	2.347	2.294 × 10^−2^

**Table 4:** Numerical analysis results of PES/HNT and PVDF/HNT membranes in different geometries.

		PES/HNT			PVDF/HNT	
	Equivalent Stress (N/mm^2^)	Displacement (mm)	In-Plane Principal Strain	Equivalent Stress (N/mm^2^)	Displacement (mm)	In-Plane Principal Strain
Hexagon	5.704 × 10^−1^	5.042	1.947 × 10^−2^	6.185 × 10^−1^	4.643	1.680 × 10^−2^
Circle	4.772 × 10^−1^	5.134	1.764 × 10^−2^	5.138 × 10^−1^	4.731	1.539 × 10^−2^
Square	5.838 × 10^−1^	4.800	2.122 × 10^−2^	6.329 × 10^−1^	4.417	1.837 × 10^−2^
Ellipse 1.25	5.154 × 10^−1^	4.982	2.028 × 10^−2^	5.558 × 10^−1^	4.589	1.775 × 10^−2^
Ellipse 1.5	5.242 × 10^−1^	4.672	2.169 × 10^−2^	5.668 × 10^−1^	4.300	1.908 × 10^−2^
Ellipse 1.75	5.257 × 10^−1^	4.331	2.251 × 10^−2^	5.687 × 10^−1^	3.981	1.990 × 10^−2^
Ellipse 2	5.197 × 10^−1^	4.010	2.303 × 10^−2^	5.627 × 10^−1^	3.681	2.041 × 10^−2^
Ellipse 2.25	5.152 × 10^−1^	3.720	2.336 × 10^−2^	5.579 × 10^−1^	3.410	2.073 × 10^−2^
Ellipse 2.5	5.044 × 10^−1^	3.465	2.356 × 10^−2^	5.464 × 10^−1^	3.170	2.092 × 10^−2^
Ellipse 2.75	4.962 × 10^−1^	3.238	2.368 × 10^−2^	5.377 × 10^−1^	2.958	2.104 × 10^−2^
Ellipse 3	4.871 × 10^−1^	3.037	2.376 × 10^−2^	5.279 × 10^−1^	2.769	2.111 × 10^−2^
Ellipse 3.25	4.787 × 10^−1^	2.859	2.381 × 10^−2^	5.188 × 10^−1^	2.601	2.116 × 10^−2^
Ellipse 3.5	4.703 × 10^−1^	2.697	2.383 × 10^−2^	5.095 × 10^−1^	2.450	2.116 × 10^−2^
Ellipse 3.75	4.617 × 10^−1^	2.552	2.382 × 10^−2^	5.003 × 10^−1^	2.313	2.114 × 10^−2^
Ellipse 4	4.551 × 10^−1^	2.419	2.379 × 10^−2^	4.931 × 10^−1^	2.187	2.109 × 10^−2^

**Table 5:** Numerical analysis results of PES/SiO_2_ and PVDF/SiO_2_ membranes in different geometries.

		PES/SiO_2_			PVDF/SiO_2_	
	Equivalent Stress (N/mm^2^)	Displacement (mm)	In-Plane Principal Strain	Equivalent Stress (N/mm^2^)	Displacement (mm)	In-Plane Principal Strain
Hexagon	5.511 × 10^−1^	5.219	2.076 × 10^−2^	5.905 × 10^−1^	4.868	1.828 × 10^−2^
Circle	4.625 × 10^−1^	5.314	1.870 × 10^−2^	4.925 × 10^−1^	4.958	1.665 × 10^−2^
Square	5.642 × 10^−1^	4.970	2.257 × 10^−2^	6.043 × 10^−1^	4.633	1.993 × 10^−2^
Ellipse 1.25	4.992 × 10^−1^	5.158	2.147 × 10^−2^	5.323 × 10^−1^	4.811	1.916 × 10^−2^
Ellipse 1.5	5.071 × 10^−1^	4.838	2.293 × 10^−2^	5.420 × 10^−1^	4.510	2.053 × 10^−2^
Ellipse 1.75	5.084 × 10^−1^	4.487	2.374 × 10^−2^	5.437 × 10^−1^	4.179	2.134 × 10^−2^
Ellipse 2	5.025 × 10^−1^	4.157	2.426 × 10^−2^	5.377 × 10^−1^	3.867	2.187 × 10^−2^
Ellipse 2.25	4.981 × 10^−1^	3.859	2.459 × 10^−2^	5.331 × 10^−1^	3.585	2.219 × 10^−2^
Ellipse 2.5	4.876 × 10^−1^	3.596	2.479 × 10^−2^	5.219 × 10^−1^	3.336	2.239 × 10^−2^
Ellipse 2.75	4.795 × 10^−1^	3.363	2.491 × 10^−2^	5.135 × 10^−1^	3.116	2.251 × 10^−2^
Ellipse 3	4.708 × 10^−1^	3.157	2.499 × 10^−2^	5.042 × 10^−1^	2.921	2.259 × 10^−2^
Ellipse 3.25	4.626 × 10^−1^	2.973	2.504 × 10^−2^	4.954 × 10^−1^	2.747	2.264 × 10^−2^
Ellipse 3.5	4.546 × 10^−1^	2.807	2.505 × 10^−2^	4.867 × 10^−1^	2.589	2.265 × 10^−2^
Ellipse 3.75	4.462 × 10^−1^	2.658	2.505 × 10^−2^	4.779 × 10^−1^	2.448	2.264 × 10^−2^
Ellipse 4	4.399 × 10^−1^	2.521	2.503 × 10^−2^	4.710 × 10^−1^	2.318	2.260 × 10^−2^
